# How do popularity cues drive impulse purchase in live streaming commerce? The moderating role of perceived power

**DOI:** 10.3389/fpsyg.2022.948634

**Published:** 2022-08-04

**Authors:** Liguo Lou, Yongbing Jiao, Myung-Soo Jo, Joon Koh

**Affiliations:** ^1^School of Economics and Management, Ningbo University of Technology, Ningbo, China; ^2^Business School, Taizhou University, Taizhou, China; ^3^Desautels Faculty of Management, McGill University, Montreal, QC, Canada; ^4^Department of Business Administration, Chonnam National University, Gwangju, South Korea

**Keywords:** live streaming commerce, popularity cues, impulse purchase, perceived power, perceived streamer reputation, perceived competition

## Abstract

A significant characteristic of live streaming commerce is that popularity cues are tactically created and utilized to improve product sales, as atmospheric cues. However, research on live streaming commerce that investigates the effects of popularity cues is scarce. This study aims to reveal the role of popularity cues, including streamer popularity and product popularity, in promoting consumers’ impulse purchase. Following the stimulus–organism–response paradigm, this study reveals the underlying mechanism. This study surveyed 402 customers and empirically demonstrates that streamer popularity and product popularity can trigger consumers’ impulse purchase by enhancing perceived streamer reputation and perceived competition, respectively. Meanwhile, perceived power, as an inherent factor of consumers, plays a moderating role that only attenuates the effect of streamer popularity on perceived streamer reputation. This study contributes to a better understanding of the working mechanism of popularity cues and offers practical insights into how to effectively utilize these atmospheric cues in live streaming commerce.

## Introduction

Live streaming, as a new technology that enables users to interact with each other over the internet in real time, has been used to facilitate streamers (broadcasters) to fully display and introduce products to persuade consumers—this is termed live streaming commerce (Park and Lin, 2020; Lu and Chen, 2021). Most prominent e-commerce (e.g., Taobao) and social media (e.g., Facebook) platforms have adopted such live streaming commerce, in an effort to expand their reach and improve their business performance. Compared with traditional e-commerce, live streaming commerce has two unique characteristics: (1) streamers interact closely with consumers (viewers) by showing different characteristics of products, answering customer questions in real time, and organizing live activities that entertain and encourage customers to buy on the spot (Sun et al., 2019; Wongkitrungrueng and Assarut, 2020); and (2) consumers can more readily interact with streamers through a public scrolling text screen, by asking questions, liking, commenting, or even rewarding the streamer with virtual gifts (Li R. et al., 2021; Li Y. et al., 2021). These significant advantages have made live streaming commerce a mainstream online shopping channel. According to eMarketer (2021), China has 824.5 million online shopping consumers, of which more than 371 million will make at least one purchase from a live streaming commerce platform by the end of 2023. Another industrial report published by Statista (2021) reveals that the live streaming commerce has exploded in China with sales revenue expected to reach 3.5 trillion Yuan by the end of 2022.

In line with its growing use in practice, live streaming commerce is gaining substantial research attention, in efforts to explore how it works (e.g., Sun et al., 2019; Park and Lin, 2020; Wongkitrungrueng and Assarut, 2020; Kang et al., 2021; Lu and Chen, 2021; Zhang et al., 2022). Considering its relative newness though, this research stream still lacks evidence related to several relevant topics. In particular, a prominent feature of live streaming commerce is the presence of visible, real-time information about the number of viewers, viewers’ engagement behaviors (i.e., liking, commenting, sharing, subscribing, or rewarding), and product sales information on a scrolling text screen (Fei et al., 2021). Such information only appears because the marketers responsible for the live streaming provide it. Why might they do so? This study posits that marketers send such signals, which relate to both the streamer and the product, to stimulate internal and behavioral reactions among viewers (consumers). In particular, these data could provide signals of streamer popularity and product popularity (Jin and Phua, 2014; He and Oppewal, 2018; Kao et al., 2021). Popularity signals in turn might evoke impulsivity, which could have beneficial or detrimental effects. According to prior research (Liu et al., 2013; Chan et al., 2017), approximately 40% of all online consumer expenditure is attributed to impulse purchase, which suggests that online impulse purchase has become an epidemic. Since impulse purchase contributes significantly to firms’ product sales (Jeffrey and Hodge, 2007; Lo et al., 2022), it is relevant, for marketing practitioners, consumers, and policy makers, to understand whether and how popularity cues might trigger consumers’ impulse purchase.

Prior impulse purchase research tends to adopt the stimulus–organism–response (S-O-R) paradigm to explain how consumer characteristics, store characteristics, product characteristics, or situational stimuli affect impulse purchase (e.g., Liu et al., 2013; Chan et al., 2017; Wu et al., 2021; Lo et al., 2022). By applying this paradigm, this study predicts that streamer popularity and product popularity represent situational and environmental stimuli, associated with live streaming commerce. Regarding the organism-related factors, this study suggests that consumer perceived streamer reputation and perceived competition could duly represent consumers’ internal states while encountering such popularity cues, respectively. Meanwhile, Wells et al. (2011) and Chan et al. (2017) suggest that consumer characteristics might moderate the effects of situational stimuli on consumers’ reactions. Thus, to maximize the significant benefits that popularity cues can offer, it is necessary to uncover which factor of consumer characteristics has potential interaction effects with popularity cues. As a ubiquitous, inherent characteristic, this study posits that consumers’ perceptions of their own power might influence their individual thoughts, feelings, and actions in response to social and environmental stimuli (Anderson et al., 2012; Jiang et al., 2018; Wei et al., 2020). Specifically, this study investigates whether consumers’ perceived power interacts with popularity cues to influence consumers’ internal states, as a boundary condition. To do so, this study (1) identifies cues of streamer popularity and product popularity; (2) investigates how streamer popularity and product popularity might trigger impulse purchase through enhanced perceived streamer reputation and perceived competition; and (3) verifies a moderating role of consumers’ perceived power. In addition to expanding the nascent stream of live streaming commerce literature, by integrating the impacts of popularity cues, this study thus offers insights to help firms, streamers, and consumers to use live streaming commerce effectively.

## Literature review

### Live streaming commerce

Through the integration of traditional e-commerce and streaming technology, live streaming commerce provides consumers with richer interactions in online shopping experience. Its rapid growth became especially intense during the COVID-19 pandemic (Zhang et al., 2022). In a sense, live streaming commerce comprises two modes: e-commerce integrated into live streaming and live streaming embedded in e-commerce (Lu and Chen, 2021). Existing research has focused on these two modes and explored their working mechanisms from different perspectives. To better understand the academic community’s impetus regarding live streaming commerce research, this study systematically reviews the relevant literature, whereof the similarities and differences are summarized in Table 1.

**TABLE 1:** Summary of related research in live streaming commerce.

Study	Theory	Model summary				Data source(s)	Major finding(s)
		Independent variable(s)	Mediator(s)	Dependent variable(s)	Moderator(s)		
Sun et al. (2019)	IT affordance perspective	Visibility, Metavoicing, Guidance shopping	Immersion, Presence	Purchase intention	–	Taobao Live, JD Live, Mogujie Live, Sina Weibo Live	Visibility, metavoicing, and guidance shopping enhance consumers’ purchase intentions through immersion and presence.
Park and Lin (2020)	Fit theory	Wanghong (source)-product fit, Live content-product fit, Self-product fit	Wanghong trustworthiness, Wanghong attractiveness, Utilitarian attitude, Hedonic attitude	Intention to buy	–	Taobao Live	Source-product fit affects perceived source attractiveness and trustworthiness; product-content fit affects utilitarian and hedonic attitudes toward the content. Source trustworthiness, hedonic attitude, and self-product fit increase intention to buy.
Wongkitrungrueng and Assarut (2020)	Value theory, Trust theory	Utilitarian value, Hedonic value, Symbolic value	Trust in products, Trust in sellers	Customer engagement	–	Facebook Live	Symbolic value has direct and indirect effects on customer engagement through trust in sellers; utilitarian and hedonic values have indirect effects on customer engagement through trust in products and trust in sellers.
Gao et al. (2021)	ELM	Central route factors (e.g., information completeness), Peripheral route factors (e.g., streamer attractiveness)	Perceived persuasiveness	Purchase intention, Response intention	Mindfulness	Taobao Live, JD Live, Tiktok Live	Both route factors exert significant effects on viewers’ perceptions of the persuasive message and then lead to purchase and response intentions. Mindfulness positively moderates the relationship between perceived persuasiveness and response intention.
Kang et al. (2021)	S-O-R framework	Interactivity (responsiveness, personalization)	Tie strength	Customer engagement behavior	Popularity, Tenure of membership	Sina Weibo Live	Responsiveness and personalization have inverted U-shaped relationships with tie strength and customer engagement behavior. Membership tenure and popularity moderate (strengthen and weaken, respectively) the relationships between interactivity and tie strength.
Li Y. et al. (2021)	Socio-technical perspective, Attachment theory	Interaction, Identification, Synchronicity, Vicarious expression	Emotional attachment to streamers, Platform attachment	Visit duration, User retention	–	Taobao Live	Interaction, identification, synchronicity, and vicarious expression positively affect emotional attachment to streamers and platform attachment, which increase user stickiness.
Lu and Chen (2021)	Uncertainty reduction perspective	Physical characteristic similarity, Value similarity	Product fit uncertainty, product quality uncertainty, Trust	Purchase intention	–	Taobao Live, JD Live, Douyin Live, Kuaishou Live	Streamers’ physical characteristic similarity enhances consumers’ purchase intention through decreasing product fit uncertainty. Streamers’ value similarity enhances consumer trust, which then decreases product fit uncertainty and product quality uncertainty and ultimately promotes purchase intention.
Guo et al. (2022)	Source characteristic perspective	Beauty, Warmth, Expertise, Humor, Passion	Perceived utilitarian value, Perceived hedonic value, Streamer popularity	Watching intention, Purchase intention	–	Taobao Live, JD Live, Douyin Live, Kuaishou Live	Beauty, expertise, humor, and passion enhance perceived hedonic value; both warmth and expertise increase perceived utilitarian value. Perceived utilitarian value and perceived hedonic value promote watching and purchase intentions. Perceived hedonic value increases streamer popularity.
Zhang et al. (2022)	Socio-technical perspective	Active control, Synchronicity, Two-way communication, Personalization, Visibility	Trust in streamers, Trust in products	Continuance intention	Live streaming genre	Taobao Live	Active control, two-way communication, synchronicity, personalization, and visibility enhance trust, which increases continuance intentions. In addition, live streaming genre moderates the impacts of trust on continuance intention.
This study	S-O-R framework	Streamer popularity, Product popularity	Perceived streamer reputation, Perceived competition	Impulse purchase	Perceived power	Taobao Live	Streamer popularity and product popularity can trigger consumers’ impulse purchase by enhancing perceived streamer reputation and perceived competition, respectively. Consumers’ perceived personal power attenuates such relationships.

Live streaming commerce research is necessarily interdisciplinary. The development of technology is a prerequisite of live streaming commerce. Thus, some prior studies have focused on the technical perspectives to reveal the working mechanism of live streaming commerce. For example, Sun et al. (2019) adopt the information technology affordance theory to demonstrate the effects of technical characteristics on consumers’ purchase intention. Kang et al. (2021) focus on the technology-enabled interactivity characteristics of live streaming and confirm its effects on customer engagement behavior, based on the S-O-R paradigm. Li Y. et al. (2021) and Zhang et al. (2022) further adopt the socio-technical perspective to investigate the impacts of social and technical characteristics on consumers’ stickiness and continuance intention.

Meanwhile, to optimize the marketing strategies of live streaming commerce, some prior studies have concentrated on the impacts of marketing stimuli on consumers’ internal and behavioral reactions. For instance, Wongkitrungrueng and Assarut (2020) integrate the value and trust theories to examine the effects of customer value on customer trust and customer engagement. Park and Lin (2020) use the fit theory to investigate the effects of keeping fit between source and product, content and product, as well as self and product on consumers’ internal evaluation and intention to buy. Gao et al. (2021) apply the elaboration likelihood model (ELM) to explain the central and peripheral factors that influence consumer perceived persuasiveness and behavioral response. Further, Lu and Chen (2021) and Guo et al. (2022) reveal the impacts of streamers’ characteristics on consumers’ psychological and behavioral responses from the uncertainty reduction perspective and source characteristic perspective, respectively.

Previous research presented in Table 1 reveals that technology- and marketing-related characteristics can lead live streaming commerce to become a mainstream online shopping channel. Given the importance of live streaming in promoting consumption, this study further explores the working mechanism of live streaming commerce from a new perspective. Table 1 shows that the current study differs from prior research in the following three aspects: (1) as the live streaming commerce platform is especially adept at creating and sending popularity signals to facilitate streamers to introduce products, this study focuses on streamer popularity and product popularity as marketing stimuli and aims to explore their effectiveness; (2) considering that prior studies have not yet explored the impulse purchase issue in the context of live streaming commerce, this study attempts to link the relationships between popularity cues and consumers’ impulse purchase; and (3) consumers’ perceptions of their own power influence their information processing, this study therefore intends to examine whether the high (low) degree of power perceptions could enable consumers to make judgment relying less (more) on popularity cues.

### Popularity cues

Marketers are adept in creating and communicating popularity cues to exert influences on consumers’ evaluations and behaviors. Dean (1999) proposes that brand popularity with third-party endorsement and event sponsorship are three major marketing cues designed to positively influence consumers’ brand perceptions. A brand’s product market share is referred to brand popularity that signals a product having superior quality and carrying a high value for consumers, following the signal theory (Dean, 1999; van Herpen et al., 2009). Similarly, Magnini et al. (2013) and Kim (2018) define brand popularity as the degree to which a brand is widely bought by consumers, and they propose that using the popularity cue (e.g., No. 1 in sales) in advertisement could enhance consumers’ brand evaluations.

In the social media marketing field, an increasing number of research has addressed the role of popularity cues, wherein popularity cues are divided into three components, namely, product popularity, celebrity (streamer) popularity, and post (content) popularity. With respect to product popularity, it plays an increasingly important role in consumers’ purchase decisions because most consumers are affected by how other consumers think and feel about a product in the online shopping context (Ahn, 2007). Luo et al. (2014) define deal popularity as “the visually displayed information of the cumulative number of deals sold to other consumers” (p. 20), and further propose that deal popularity could influence a focal consumer’s purchase through signaling the deal worth. Mou and Shin (2018) use the term “social popularity” to refer to the degree of consumers liking or purchasing a product and confirm that social popularity could enhance consumer trust, perceived product quality, and perceived value. In addition, Kao et al. (2021) find interesting results that while high online deal popularity could increase individualistic (Australian) consumers’ psychology risk, which in turn lowers their purchase intention, it could decrease collectivistic (Taiwan) consumers’ risk perception, which then enhances their purchase intention. Celebrity popularity refers to the number of followers a celebrity has, which has the potential to improve source credibility, social identification, and buying intention (Jin and Phua, 2014). Ladhari et al. (2020) demonstrate that homophily and emotional attachment have positive effects on vloggers’ popularity, which in turn enhances viewers’ purchase intention. With regard to post popularity, the number of likes and comments could be indicators of post popularity, whereof the higher degree suggests that brand fans share more enthusiasm about the brand (Swani and Milne, 2017). Chang et al. (2015) confirm that post popularity could lead to users’ usefulness perception of and preference for posts, which then enhances users’ contribution behaviors. Similarly, Yang et al. (2020) demonstrate that post popularity could enhance consumer trust, which in turn promotes consumer attitude and purchase intention.

The popularity cue studied in prior research includes many typologies depending on the research setting. Notwithstanding the important role of popularity cues, they have not been given much attention in live streaming commerce research. Given that streamer popularity and product popularity cues are the most visible stimuli in the live streaming commerce (Fei et al., 2021), they are worth investigating to achieve a better understanding of the occurrence of impulse purchase.

### Impulse purchase

Impulse purchase refers to consumers’ unplanned, compelling, and hedonically complex product buying behavior, whereby consumers fail to carefully consider all the relevant information and quickly make purchase decisions (Stern, 1962; Rook, 1987; Chen et al., 2019; Gulfraz et al., 2022). Impulse purchase was first studied in the brick-and-mortar store consumption context. Kotler (1973) proposes a concept of atmospherics, implying that marketers could promote consumers’ purchase via the creation of a suitable shopping environment and atmosphere. Based on this foundation, psychology and consumer behavior researchers have arrived at a consensus on impulse purchase via suggesting that in-store stimuli could generate consumers’ impulse purchase. For example, discount promotion (Blattberg et al., 1995), background music (Milliman, 1982), and olfactory cues (Spangenberg et al., 1996) could induce impulse purchase via affecting consumers’ internal reactions.

With the tremendous growth of e-commerce, it is quite common for consumers to perform online impulse purchase because shopping online frees consumers from the constraints that they might experience in physical stores (Liu et al., 2013; Huang, 2016; Chan et al., 2017; Andronie et al., 2021). On the one hand, technology-related website cues are important antecedents of impulse purchase. For example, Koufaris (2002) and Wu et al. (2016) integrate the technology acceptance model and flow theory to explain how online impulse purchase occurs. Meanwhile, website characteristics, including task- and mood-relevant cues could stimulate consumers’ internal reactions and impulse purchase (Parboteeah et al., 2009). Similarly, Liu et al. (2013) adopt the S-O-R paradigm to examine the effects of website attributes (i.e., product availability, visual appeal, and website ease of use) on consumers’ internal evaluations and impulse purchase. One the other hand, marketing-related cues could also lead to impulse purchase. For instance, the information quality of advertisement and the number of “Like” in Chen et al. (2016); recommender- and product-related signals in Chen et al. (2019); Instagram advertisement, opinion leader, and user-generated content in Djafarova and Bowes (2021); and limited-quantity and limited-time scarcity in Wu et al. (2021) have been confirmed to have effects on consumers’ online impulse purchase.

The abovementioned studies suggest that factors related to e-commerce’s technology and marketing cues can elicit impulse purchase. As a new online shopping mode, live streaming commerce is evidently believed to be conducive to impulse purchase. As little live streaming commerce research has investigated impulse purchase (Lo et al., 2022), this study aims to reveal how consumers’ impulse purchase occurs after being exposed to streamer popularity and product popularity cues based on the S-O-R paradigm.

## Hypothesis development

### Activating internal reactions: Stimuli of popularity cues

Combining the definitions of popularity in prior research with live streaming commerce characteristics, this study identifies popularity cues that include streamer popularity and product popularity. Streamer popularity refers to the visually displayed information of consumers’ behaviors of positively interacting with a streamer, such as viewing, liking, commenting, sharing, subscribing, rewarding, and so on. Product popularity refers to the visually displayed information of the cumulative number of products sold to consumers. Regarding internal reactions, this study establishes perceived streamer reputation and perceived competition as organism-related constructs. The reasons are twofold: first, the streamer in live streaming commerce has been facing increasingly fierce competition because of the increase in streamer volume, such that streamer reputation could be considered as one of the most important intangible assets for survival and success in a competitive environment (Su et al., 2016). Second, competition refers to a purchase situation where one would need to compete with other consumers to achieve the goal of buying a product (Aggarwal et al., 2011; Nichols, 2012), thereby suggesting that competition is a situation-dependent transitory state that may exist in a live streaming commerce context owing to the popularity of the product. Thus, perceived streamer reputation and perceived competition are deemed to be closely related to streamer popularity and product popularity, respectively. To establish such relationships, this study adopts signal theory as a specific theoretical foundation within the S-O-R paradigm. The signal theory is developed under the condition of asymmetric information, which addresses the role of signals sent out by one party who has information advantage in reducing market uncertainty and promoting market efficiency (Spence, 1973). Based on the signal theory, product or service providers may invest in useful signals that convey some meaningful and relevant information (cues) to consumers to affect their internal states, consequently facilitating transactions (Chen et al., 2019; Yang et al., 2020; Jang and Chung, 2021).

The reputation concept has been well developed in previous research. For instance, supplier reputation refers to the extent to which a supplier is honest and concerned about its customers (Doney and Cannon, 1997; Pera et al., 2016). Brand reputation refers to the extent to which a brand has the ability to provide high quality services (Sengupta et al., 2015). Based on prior research, this study defines perceived streamer reputation as consumers’ confidence level of a streamer who is honest and concerned about them. Reputation is a relative concept, and it depends on the comparison between different competitors and their performance (Deephouse and Carter, 2005). This statement suggests that the perceived streamer reputation could be enhanced by streamer popularity through two ways. First, following the signal theory, popularity cues could be used to signal that a product has superior quality (Dean, 1999; Jang and Chung, 2021), a deal is worthy (Luo et al., 2014), and a brand post is useful (Chang et al., 2015). In the same way, streamer popularity could signal that a streamer has an ability and willingness to serve consumers well to promote their shopping performance, which has the potential to enhance consumer perceived streamer reputation. Second, popularity cues could be represented by consumers widely buying a brand (Magnini et al., 2013; Kim, 2018) and consumers positively rating a product (Ahn, 2007; Mou and Shin, 2018), suggesting that a brand or product is more superior than competitor brands or products. Similarly, a streamer with a high level of popularity indicates that they are more superior than other streamers, which is likely to make consumers perceive more reputation. In addition, popularity cues are confirmed to improve consumer trust (Jin and Phua, 2014; Yang et al., 2020), and consumer trust is demonstrated to have a positive effect on reputation perception (Singh et al., 2020). Therefore, streamer popularity has the potential to enhance consumers’ reputation perception. Taken together, this study proposes the relationship between streamer popularity and perceived streamer reputation as follows:

**Hypothesis 1**: Streamer popularity has a positive effect on perceived streamer reputation.

Following Nichols (2012), perceived competition refers to one’s belief that one would need to compete with other buyers to achieve a goal of buying products in the live streaming commerce situation. According to the signal theory (Dean, 1999; He and Oppewal, 2018), the product popularity can be a diagnostic cue that influences consumer perceived competition through indirect and direct ways. Regarding the indirect way, product popularity can signal a product with superior quality and value for consumers (Dean, 1999; Luo et al., 2014; Jang and Chung, 2021), and it has the potential to not only make consumers believe this product is worth buying but also stimulate them to infer that others would also want to buy it. In other words, the product popularity cue can be referred as a demand-based cue indicating high current or expected demand for a high-quality product with superior value, leading to competitive consumption (Teubner and Graul, 2020). Concerning the direct way, product popularity, as the term suggests, signals that a product is liked and widely bought by many consumers (Mou and Shin, 2018; Kao et al., 2021). Consequently, product popularity, which provides social validation for many consumers buying the same product, is likely to stimulate a consumer to perceive that they are endeavoring to gain what others are attempting to gain simultaneously. Accordingly, this study has the following hypothesis:

**Hypothesis 2**: Product popularity has a positive effect on perceived competition.

### Effects of perceived streamer reputation and perceived competition on impulse purchase

Chaiken (1980) proposes a heuristic vs. systematic information processing model for decision-making, in which the systematic information processing involves detailed assessments of information and related cognitions, and the heuristic information processing instead avoids detailed analyses and relies on simple rules. From this perspective, impulse purchase likely reflects heuristic information processing, because this purchase process is simple and involves little cognitive effort (Stern, 1962; Gulfraz et al., 2022; Lo et al., 2022). Chen et al. (2016, 2019) reveal that advertising information quality and interpersonal trust can provide heuristic cues that minimize consumers’ cognitive decision-making efforts and trigger more impulse purchases online. Because impulse purchase is characterized by a lack of cognitive deliberation (Rook, 1987; Chan et al., 2017; Chen et al., 2019), this study proposes that the effects of perceived streamer popularity and perceived competition on impulse purchase might be explained by heuristic information processing theory.

Online seller reputation is a strategic resource for consumers toward reducing concerns and uncertainty (Karimov and Brengman, 2014) and building trust (Meilatinova, 2021). Accordingly, a streamer who has high reputation is generally considered to be reliable and honest. Meanwhile, as Chaiken (1980) suggests, when people employ a heuristic information processing strategy, source characteristics might generate greater impacts on persuasion than information characteristics. Combining these logics, when consumers perceive a streamer to have good reputation, they may relinquish the thoughtful process of deliberating product-related information and instead thoughtlessly decide to buy a product by trusting the streamer. In other words, perceived streamer reputation can help consumers reduce the amount of cognitive effort required and simplify the decision-making process, consequently facilitating the occurrence of impulse purchase. Accordingly, this study has the following hypothesis:

**Hypothesis 3**: Perceived streamer reputation has a positive effect on impulse purchase.

The presence of rivalry, scarcity, and win–lose performance anxiety are three major elements of competition (Nichols, 2012). While little research has addressed the effects of competition on consumers’ behavior, extensive research has explored the impacts of rivalry, scarcity, and performance anxiety information on consumers’ reactions. For example, scarcity information has been confirmed to raise the urgency of buying because it could lead consumers to employ a heuristic information processing strategy rather than the systematic information processing strategy to make a quick judgment (Aggarwal et al., 2011; Lo et al., 2022). Furthermore, limited-quantity scarcity accompanied by perceived rivalry could make consumers feel that they are in direct competition with other consumers, thereby making an impulse purchase decision simply and immediately under pressure (Wu et al., 2021). Meanwhile, time restrictions could make consumers experience a performance anxiety about wasting opportunities, whereof a possible consequence is buying relevant products impulsively (Swain et al., 2006). Based on the heuristic information processing theory and relevant prior studies, this study posits that perceived competition is likely to increase a sense of urgency which impedes consumers’ cognitive decision-making. Accordingly, this study proposes the following hypothesis regarding the relationship between perceived competition and impulse purchase:

**Hypothesis 4**: Perceived competition has a positive effect on impulse purchase.

### Moderating role of perceived power

Power considerations are ubiquitous and closely related to one’s social status, education, income, age, gender, and so on. Galinsky et al. (2003) propose that power is a psychological state, namely, a perception of one’s capability to influence others because of the control over resources or social position. Correspondingly, perceived power is defined as “the perception of one’s ability to influence another person or other people” (Anderson et al., 2012, p. 316). Prior research has increasingly focused on the role of power in affecting consumers’ reactions and suggests that high power fosters an agentic orientation, which is associated with an increased need for control, dominant behaviors, and independence; conversely, low power is linked to a communal orientation reflecting submissive behaviors, lack of control, and dependence on others (Rucker et al., 2012; Wongkitrungrueng et al., 2018). On this foundation, Bellezza et al. (2013) confirm that high-power consumers are more capable of behaving as they deem appropriate and are less affected by other people’s judgments. Further, Lee et al. (2020) demonstrate that high-power consumers are more likely to use tipping as a monitoring system based on service quality received from a server rather than image protection.

The aforementioned prior research contributes toward expounding the interaction effects of perceived power and popularity cues on consumers’ internal reactions in the current study. On the one hand, considering the nature of streamer popularity and product popularity, they respectively reflect other consumers’ supports and preferences for the streamer and product, thereby embodying forms of social influence (Mou and Shin, 2018; Guo et al., 2022). As people with more power have more resources, they tend to be more dominant and less concerned with other people’s judgments (Rucker et al., 2012; Bellezza et al., 2013; Lee et al., 2020). Based on these statements, consumers who perceive more power will perceive less streamer reputation and competition when viewing the streamer popularity and product popularity cues because they seldom make judgments depending on social cues. On the other hand, power reflets an individual’s capability, which helps individuals make judgments following their own experience and knowledge rather than third-party and contextual information (Whitson et al., 2013; Wei et al., 2020). Taken together, this study puts forth the following hypotheses regarding the moderating role of perceived power:

**Hypothesis 5**: The relationship between streamer popularity and perceived streamer reputation is weaker for consumers with high perceived power than it is for consumers with low perceived power.

**Hypothesis 6**: The relationship between product popularity and perceived competition is weaker for consumers with high perceived power than it is for consumers with low perceived power.

**FIGURE 1:**
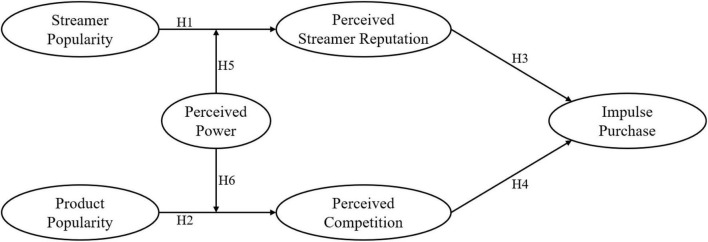
Research model.

This study seeks to reveal the working mechanism of popularity cues in a live streaming commerce context, grounded in the S-O-R paradigm. Figure 1 depicts the conceptual model for this study.

## Research method

### Measurement

This study conducts an empirical study using an online survey method for data collection and hypothesis test. The unit of analysis is at the individual level (i.e., consumers who view live streaming and purchase products impulsively). A survey instrument is designed to obtain data on six research variables. Measurement items are drawn from prior studies and slightly modified to ensure their appropriateness for this study. All constructs are measured with multiple items based on a seven-point Likert scale (1 = strongly disagree, 7 = strongly agree). The measurement items and related sources are presented in Table 2.

**TABLE 2:** Measurement items for the constructs.

Constructs	Items	Sources
Streamer popularity	1. This streamer has many fans.	Jin and Phua, 2014;
	2. Many people list this streamer.	Guo et al., 2022
	3. There are many audiences watching this streamer.	
	4. Based on the number of fans, this streamer is popular.	
	5. Based on the number of audience, this streamer is popular.	
Product popularity	1. This product is popular.	van Herpen et al., 2009
	2. This product is sold well.	
	3. Many people want to buy this product.	
Perceived streamer reputation	1. This streamer has a reputation for being honest.	Doney and Cannon, 1997; Su et al., 2016
	2. This streamer is known to be concerned about customers.	
	3. This streamer has a good reputation.	
	4. This streamer is a successful person.	
Perceived competition	1. I will have to compete with others to buy this product.	Nichols, 2012
	2. I will be seeking out something that others are also seeking out.	
	3. Other potential buyers are “rivals” of mine.	
	4. If I am able to buy this product, it means that I “won.”	
	5. Trying to buy this product means a competition.	
	6. It means I succeeded if I am able to buy this product.	
Perceived power	1. I think I have a great deal of power.	Anderson et al., 2012
	2. I can get others to listen to what I say.	
	3. I can get others to do what I want.	
	4. If I want to, I can make the decisions.	
Impulse purchase	1. My purchase is spontaneous.	Huang, 2016
	2. My purchase is unplanned.	
	3. I did not intend to do this purchase before this shopping trip.	
	4. I could not resist to do this purchase.	

### Data collection

Regarding the data collection source of live streaming commerce research, Table 1 shows that some research collect data from one platform, while some obtain data from more than one platform. As each platform has its unique characteristics, consumers may experience different stimuli of popularity cues; therefore, this study establishes Taobao Live as the research setting. Taobao Live is the leading live streaming commerce platform in China, wherein a large number of streamers endeavor to introduce a variety of clothing, cosmetics, food, electronic products, and so on, suggesting an intense competition.

This study utilizes the Sojump data gathering platform^1^ to implement online surveys. A Web link of the Chinese questionnaire is forwarded to potential respondents via WeChat, one of the most popular social media in China. The data collection is conducted from November 2020 to December 2020. To ensure that the respondents have the experience of watching Taobao Live and buying products, this study adds three screening questions (i.e., Have you had the experience of watching Taobao Live recently? Which streamer did you view? Have you had the experience of buying a product while watching the live streaming?). The respondents are first asked to answer these three questions. If they cannot answer them, they have to terminate the questionnaire. The respondents who have answered the abovementioned questions are asked to complete the questionnaire. Within a month, a total of 438 questionnaires are collected, whereof 36 questionnaires are excluded due to incomplete response with missing the three screening questions or aberrant responses lacking justification. Consequently, 402 valid samples are collected and subsequently analyzed.

Table 3 displays the demographic statistics of respondents in detail. Specially, regarding the gender of respondents, 32.8% are male and 67.2% are female, which is similar to Sun et al. (2019); Gao et al. (2021), and Li Y. et al. (2021), confirming that females account for the vast majority in live streaming commerce.

**TABLE 3:** Demographics of respondents (*n* = 402).

Category		Frequency	Percent (%)
Gender	Male	132	32.8
	Female	270	67.2
Age	<20	52	12.9
	20–29	298	74.1
	30–39	49	12.2
	>39	3	0.7
Education	High school or lower	26	6.5
	Bachelor’s or college degree	325	80.9
	Graduate degree	51	12.7
Income (Monthly, CNY)	<5,000	293	72.9
	5,001–10,000	79	19.7
	10,001–15,000	12	3.0
	15,001–20,000	7	1.7
	>20,000	11	2.7
Most frequently viewed streamers	Jiaqi Li	106	26.4
	Viya	89	22.1
	Others	207	51.5
Total	–	402	100

## Data analysis and results

### Statistical analysis technique

Partial least squares structural equation modeling is not only suitable for handling non-normally distributed data (Chin et al., 2003), but also duly accommodates samples smaller than 500 (Hair et al., 2014). Therefore, this study decides to employ Smart PLS 3.0 to assess reliability, convergent validity, and discriminant validity of the constructs, as well as to test the hypotheses.

### Reliability and validity

A confirmatory factor analysis (CFA) is performed to investigate the reliability and validity. Table 4 shows the CFA results. According to Fornell and Larcker (1981), the Cronbach’s α value and the composite reliability (CR) value for all constructs are higher than the threshold value of 0.7, which suggest acceptable internal consistency and scale reliability. Regarding convergent validity, following Fornell and Larcker (1981), this study confirms that the standardized factor loadings of indicators for all constructs are significantly greater than 0.7, and the values of average variance extracted (AVE) for all the constructs exceed the recommended minimum of 0.5, suggesting that the convergent validity is acceptable.

**TABLE 4:** Results of reliability and convergent validity tests.

Construct	Indicators	Standardized factor loadings	Cronbach’s α	CR	AVE
Streamer popularity	SP1	0.920	0.950	0.962	0.834
	SP2	0.877			
	SP3	0.912			
	SP4	0.926			
	SP5	0.931			
Product popularity	PP1	0.861	0.854	0.911	0.773
	PP2	0.882			
	PP3	0.895			
Perceived streamer reputation*	PSR1	0.960	0.897	0.936	0.830
	PSR2	0.942			
	PSR3	0.930			
Perceived competition	PC1	0.856	0.946	0.957	0.788
	PC2	0.813			
	PC3	0.918			
	PC4	0.919			
	PC5	0.934			
	PC6	0.881			
Perceived power	PPow1	0.835	0.869	0.908	0.714
	PPow2	0.919			
	PPow3	0.885			
	PPow4	0.729			
Impulse purchase*	IP1	0.942	0.920	0.949	0.862
	IP2	0.943			
	IP3	0.899			

*The fourth item of perceived streamer reputation (PSR4) and the fourth item of impulse purchase (IP4) were deleted, on the basis of the CFA results.

Meanwhile, following Fornell and Larcker (1981), this study compares the square root of AVE for each construct with the inter-construct correlation estimates to check the discriminant validity. Table 5 reports the square roots of AVE (the diagonal elements in bold) for constructs and construct correlation estimates. Each AVE square root is greater than its corresponding row and column elements, indicating the acceptable discriminant validity of the instruments. In addition, following Hair et al. (2017), this study assesses the heterotrait–monotrait (HTMT) ratio of correlations to establish more rigorous discriminant validity. As shown in Table 6, all the HTMT values are significantly different from 1, and the largest HTMT value is 0.652, which is lower than the cut-off value of 0.90, thereby showing evidence of adequate discriminant validity.

**TABLE 5:** Results of correlation analysis and discriminant validity tests.

Construct	Mean	SD	1	2	3	4	5	6
1. Perceived competition	5.070	1.583	**0.888**					
2. Impulse purchase	4.347	1.541	0.487	**0.928**				
3. Product popularity	5.629	1.205	0.488	0.267	**0.879**			
4. Perceived power	4.848	1.165	0.368	0.399	0.339	**0.845**		
5. Streamer popularity	6.137	1.258	0.326	0.180	0.591	0.221	**0.913**	
6. Perceived streamer reputation	5.454	1.252	0.465	0.411	0.489	0.342	0.409	**0.911**

The diagonal numbers in bold are the square roots of the AVE.

**TABLE 6:** Heterotrait–monotrait (HTMT) ratio of correlations.

Construct	1	2	3	4	5	6
1. Perceived competition	–					
2. Impulse purchase	0.520	–				
3. Product popularity	0.540	0.303	–			
4. Perceived power	0.385	0.436	0.387	–		
5. Streamer popularity	0.343	0.193	0.652	0.232	–	
6. Perceived streamer reputation	0.506	0.447	0.560	0.364	0.443	–

### Common method bias test

Self-reported data from a single source may have a common method bias (CMB), which threatens the validity of the study. Therefore, this study conducts a Harman’s one factor test to verify the CMB following MacKenzie and Podsakoff (2012). The analysis results indicate that there are six latent factors exceeding 1.0 of the eigenvalues, with the first factor accounting for less than 40% of the total variance (i.e., 39.72%), suggesting that the CMB is not a significant threat in this study.

### Hypothesis test

**FIGURE 2:**
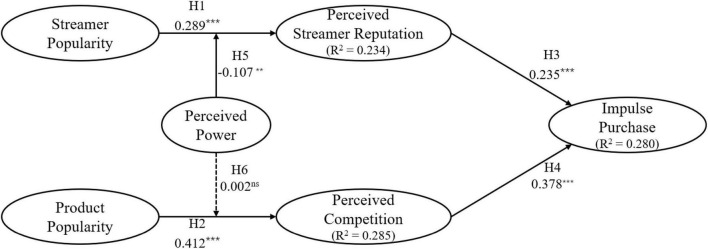
Hypothesis test results. **p < 0.01, ***p < 0.001, ns, no significant at the 5% significance level.

To test the presented hypotheses, this study utilizes Smart PLS 3.0 to perform a path analysis. The hypothesis test results are presented in Figure 2. First, regarding the relationships between stimuli and organisms, streamer popularity and product popularity are proven to have positive effects on perceived streamer reputation and perceived competition, respectively (β = 0.289, *p* < 0.001; β = 0.412, *p* < 0.001, respectively), indicating that H1 and H2 are supported. Second, with respect to the relationships between organisms and response, both perceived streamer reputation and perceived competition are confirmed to have positive effects on impulse purchase (β = 0.235, *p* < 0.001; β = 0.378, *p* < 0.001), supporting H3 and H4. Finally, regarding the moderating effect of perceived power, this study adopts the two-stage PLS approach for testing the interaction effects (i.e., streamer popularity × perceived power and product popularity × perceived power) on perceived streamer reputation and perceived competition, respectively. Results of bootstrapping on 1,000 subsamples indicate that perceived power could significantly weaken the relationship between streamer popularity and perceived streamer reputation (β = −0.107, *p* < 0.01), whereas it could not significantly weaken the relationship between product popularity and perceived competition (β = 0.002, *p* > 0.05), implying that H5 is supported while H6 is not.

This study further attempts to understand the mediator roles of perceived streamer reputation and perceived competition. Following Hair et al. (2017), this study finds that the relevant direct effects are significant (i.e., H1—H4) and the relevant indirect effects (i.e., streamer popularity → perceived streamer reputation → impulse purchase; product popularity → perceived competition → impulse purchase) are also significant (β = 0.096, *p* < 0.001; β = 0.184, *p* < 0.001, respectively) and in the same directions, thereby suggesting that perceived streamer reputation and perceived competition play partial mediator roles.

Although the focus of this study is on impulse purchase that results from the combined effects of live streaming commerce features, identifying which path (i.e., streamer popularity or product popularity) is more impactful might provide interesting insights as well. As suggested by Yi et al. (2013), this study thus compares the perceived competition → impulse purchase path (i.e., H4) with the perceived streamer reputation → impulse purchase path (i.e., H3), using Smart PLS to calculate the path difference. The findings show that the path coefficient of H4 is not significantly larger than the path coefficient of H3 (Δβ = 0.140, *p* > 0.05). That is, perceived streamer reputation and perceived competition can equivalently stimulate impulse purchase. Finally, as illustrated in Figure 2, the model explains 23.4% of the variance in perceived streamer reputation, 28.5% of the variance in perceived competition, and 28.0% of the variance in impulse purchase.

## Discussion

### Key findings

This study investigates how streamer popularity and product popularity elicit consumers’ impulse purchase through affecting consumer perceived streamer reputation and perceived competition, respectively. Additionally, the moderating role of perceived power is also examined. The results support five hypotheses and reject one, generating valuable findings and implications as presented below.

Focusing on the distinctive social cues in the context of live streaming commerce, this study develops the concepts of streamer popularity and product popularity and demonstrates their effects on consumers’ impulse purchase using a survey method, which is compared with Fei et al.’s (2021) study addressing the impacts of interaction text and herding message on live streaming commerce consumers’ purchase intention through a within-subject eye-tracking experiment. Although impulse purchase differs from rational purchase, they both contribute to improving product sales in live streaming commerce. Concerning impulse purchase, this study reveals the significant main effects of streamer popularity and product popularity via emphasizing the mediating roles of perceived streamer reputation and perceived competition, respectively. More specifically, this study testifies streamer popularity can drive impulse purchase through enhancing perceived streamer reputation, while Lo et al. (2022) indicate that streamers’ parasocial interactions can elicit impulse purchase by strengthening consumers’ affective reactions. Both the cognitive process of impulse purchase in the current study and the affective process of impulse purchase in Lo et al. (2022) show strong evidence that the streamer plays a critical role in live streaming commerce. Meanwhile, this study demonstrates that product popularity can significantly enhance perceived competition that consequently stimulates impulse purchase. Compared with previous studies (e.g., Aggarwal et al., 2011; Teubner and Graul, 2020; Wu et al., 2021) that confirm the role of creating a cue of limited-quantity products in promoting product sales, this finding stresses the role of creating a cue of a large number of potential buyers in promoting product sales. Therefore, this study also shows that the product-related factors such as product popularity can play a pivotal role in live streaming commerce.

Regarding the moderating effects of perceived power, research findings confirm that perceived power can weaken the relationship between streamer popularity and perceived streamer reputation, whereas it has no such effect on the relationship between product popularity and perceived competition. A possible explanation accounting for this interesting finding is that perceived power exists and works well in an interpersonal setting (Lee et al., 2020; Wei et al., 2020). In other words, perceived power generally comes into play during consumers’ interactions with other people, since individuals possess power aiming to control or influence others’ evaluations or actions (Anderson et al., 2012; Wongkitrungrueng et al., 2018). In this study, consumers encountering streamer popularity implies consumers’ interactions with a streamer, whereas consumers encountering product popularity refers to consumers’ interactions with a product. Considering the essential role of an individual’s power in influencing others rather than being influenced by others, consumers with high power perception are likely to produce psychological reactance when they realize a streamer is very popular, thereby leading to a lower streamer reputation evaluation. In contrast, since consumer-product interaction falls outside interpersonal setting, there might be no conditions for the functioning of perceived power, implying that perceived competition can be affected by product popularity regardless of whether consumers perceive more power or not.

### Theoretical implications

This study enriches the existing literature on live streaming commerce primarily in three ways. First, although impulse purchase is likely to occur more frequently in a live streaming context, prior research has not sufficiently addressed this issue. To fill this research gap, this study follows the S-O-R paradigm to reveal the formation mechanism of impulse purchase. As the S-O-R framework is more sophisticated than the input–output framework (Jacoby, 2002; Barbu et al., 2021), Chan et al. (2017) recommend that the S-O-R paradigm could be duly used to validate the relationships regarding marketing stimuli–internal reactions–impulse purchase. This study’s findings provide evidence that the S-O-R paradigm can work well in expounding impulse purchase in a new research context, namely, live streaming commerce. Thus, this study advances the application of the S-O-R paradigm and extends the impulse purchase research setting.

Second, following Kotler (1973), popularity cues can be considered as marketing atmospherics, which include many typologies depending on the specific marketing actions. Previous research addresses the effect of either streamer popularity (e.g., Jin and Phua, 2014; Ladhari et al., 2020) or product popularity (e.g., Mou and Shin, 2018; Kao et al., 2021) on consumers’ internal and behavioral reactions. This study advances the research on popularity cues by analyzing streamer popularity and product popularity together, based on the characteristics of live streaming commerce. Furthermore, following the signal theory (Spence, 1973; Dean, 1999) and heuristic information processing theory (Chaiken, 1980), this study demonstrates that impulse purchase can be simultaneously stimulated by emphasizing the effect of streamer popularity on perceived streamer reputation as well as highlighting the role of product popularity in promoting perceived competition. These findings provide a richer understanding that the streamer and product are both equally important components in live streaming commerce.

Third, prior research on live streaming commerce that investigates moderator(s) is limited. This study is the first, to the best of our knowledge, to adopt perceived power as a moderator to better reveal the boundary condition of popularity cues playing their roles, especially as the perceived power is ubiquitous and powerful in affecting an individual’s internal reactions (Rucker et al., 2012; Wei et al., 2020). The research findings suggest that perceived power can dampen the effect of streamer popularity on perceived streamer reputation, which provides a starting point for relevant future research avenues and consequently improves the effectiveness of creating popularity cues.

### Practical implications

This study establishes that firms can embrace live streaming to increase their business performance via enhancing consumers’ impulse purchase. Results from this study provide valuable practical implications to businesses engaged in live streaming commerce primarily in three aspects. First, streamer popularity is proven to enhance perceived streamer reputation, which in turn promotes impulse purchase. This is in consistent with Guo et al. (2022) who suggest that the streamer plays a core role in affecting consumers’ psychology and behavior. Therefore, to increase product sales, an effective approach is for firms to ask celebrities (Jin and Phua, 2014) and/or online influencers (Ladhari et al., 2020) to act the role of streamers because they have large fan bases and their reputations are readily perceived by consumers. Additionally, streamers should develop skills and capabilities to effectively interact with viewers to improve their enthusiasm, which ultimately makes them complete more engagement behaviors, such as liking, commenting, sharing, and rewarding.

Second, product popularity is demonstrated to enhance perceived competition, which then elicits impulse purchase. This emphasizes that the product is another important component in promoting live streaming commerce performance, which is similar to Park and Lin (2020) and Fei et al. (2021). Drawn from this point, this study suggests that firms should cooperate with streamers to offer products that are much more in vogue. Moreover, the frequently used price-off promotion (Blattberg et al., 1995) is suggested to be used to promote product popularity. Summarily, appropriate marketing tactics that are helpful for creating product popularity cues should be used to make consumers believe that they should compete with others to buy relevant products. It is important that marketers should strategically leverage both the streamer popularity and product popularity cues.

Third, the interaction effect of streamer popularity and perceived power on perceived streamer reputation is verified to be negative. This implies that streamer popularity could lose some effects in the high-power consumer segments. Moreover, though the firms cooperating with the top streamers (e.g., Jiaqi Li, a mega-influencer in China) can significantly promote product sales, they have to pay expensive commissions. Taken together, marketers are recommended to make different consumer segments based on analyzing consumers’ personal power in terms of consumers’ social status, income, education, age, and so on (Anderson et al., 2012; Wongkitrungrueng et al., 2018). On the basis of consumer segments, firms can ask some macro- and micro-influencers who charge considerably less to act as streamers to interact with high-power consumers, ultimately leading to greater reputation perception and more impulse purchase. By embracing the data-driven retail intelligence (Klieštik et al., 2022), firms are able to maximize the effect of streamer popularity, optimizing the investment in streamers.

### Limitations and future research directions

This study has the following limitations and directions for future research. The first limitation lies in the external validity, as the current research context is limited to Taobao Live—the best-known customer to customer e-commerce platform in China. Therefore, future research can develop this research model on other live streaming commerce platforms, such as JD Live, Tiktok Live, and Amazon Live, to cross-validate the results. Second, although CMB is not a serious issue in this study, the survey conducted online might lead to some bias. Future research that uses experiment (Jin and Phua, 2014; Wu et al., 2021), eye-tracking (Mou and Shin, 2018; Fei et al., 2021), or machine learning (Hopkins, 2022; Nica et al., 2022) is highly recommended to cross-validate the findings of this research. Third, this study does not consider the impacts of product characteristics (e.g., product involvement, product type) and other consumer characteristics (e.g., familiarity with live streaming commerce, self-construal) on consumers internal reactions and impulse purchase. To better reveal how popularity cues work, it is recommended that future research investigates the effects of such factors. Fourth, this study focuses on the cognitive process to reveal the effects of popularity cues on impulse purchase, while Guo et al. (2022) and Lo et al. (2022) suggest consumers’ affective characteristics are critical in triggering impulse purchase. In this vein, future research might apply the pleasure-arousal-dominance (PAD) model (Mehrabian and Russell, 1974) in enriching understanding of relationships between popularity cues and impulse purchase. Finally, there is an increasing trend to apply human-like artificial intelligence (AI) service agents in improving consumers’ experience quality (Pelau et al., 2021), which suggests the anthropomorphized virtual streamers can be used in the live streaming commerce. Thus, future research that investigates the impacts of virtual streamer on consumers’ psychology and behavior will be highly interesting.

## Conclusion

Given the explosive growth of live streaming commerce, it is imperative for firms and retailers to recognize this new trend of consumer shopping journey to improve business performance. Even if live streaming commerce managers are adept in creating and communicating various social cues to exert influences on consumers’ evaluations and behaviors, there is still no sufficient theoretical explanations for the effectiveness of such social cues. Following the S-O-R paradigm, this study elucidates how the streamer popularity and product popularity elicit impulse purchase by incorporating the perceived streamer reputation and perceived competition as mediators, respectively. Meanwhile, this study demonstrates that consumer perceived power can be regarded as a boundary condition, under which the streamer popularity may fail to maintain its impact. This study contributes to enriching the live streaming commerce literature and offers guidelines for marketers to achieve business goals.

## Data availability statement

The original contributions presented in this study are included in the article/supplementary material, further inquiries can be directed to the corresponding author.

## Author contributions

LL, YJ, M-SJ, and JK contributed to the conception and design of the study. LL organized the database. LL and YJ performed the statistical analysis, wrote the first draft of the manuscript and the sections of the manuscript. All authors contributed to manuscript revision, read, and approved the submitted version.

## Footnotes

1
http://www.sojump.com

## References

[B1] Aggarwal P. Jun S. Y. Hu J. H. (2011). Scarcity messages: a consumer competition perspective. *J. Advert.* 40 19–30.

[B2] Ahn H. J. (2007). Utilizing popularity characteristics for product recommendation. *Int. J. Electron. Commer.* 11 59–80. 10.2753/JEC1086-4415110203

[B3] Anderson C. John O. P. Keltner D. (2012). The personal sense of power. *J. Pers.* 80 313–344. 21446947 10.1111/j.1467-6494.2011.00734.x

[B4] Andronie M. Lãzãroiu G. Ştefãnescu R. Ionescu L. Cocoşatu M. (2021). Neuromanagement decision-making and cognitive algorithmic processes in the technological adoption of mobile commerce apps. *Oeconomia Copernicana* 12 1033–1062. 10.24136/oc.2021.034

[B5] Barbu C. M. Florea D. L. Dabija D. C. Barbu M. C. R. (2021). Customer experience in fintech. *J. Theor. Appl. Electron. Commer. Res.* 16 1415–1433.

[B6] Bellezza S. Gino F. Keinan A. (2013). The red sneakers effect: inferring status and competence from signals of nonconformity. *J. Consum. Res.* 41 35–54. 10.1086/674870

[B7] Blattberg R. C. Briesch R. Fox E. J. (1995). How promotions work. *Mark. Sci.* 14 122–132. 10.1287/mksc.14.3.G122 19642375

[B8] Chaiken S. (1980). Heuristic versus systematic information processing and the use of source versus message cues in persuasion. *J. Pers. Soc. Psychol.* 39 752–766. 10.1037/0022-3514.39.5.752

[B9] Chan T. K. H. Cheung C. M. K. Lee Z. W. Y. (2017). The state of online impulse-buying research: a literature analysis. *Inf. Manage.* 54 204–217.

[B10] Chang Y.-T. Yu H. Lu H.-P. (2015). Persuasive messages, popularity cohesion, and message diffusion in social media marketing. *J. Bus. Res.* 68 777–782. 10.1016/j.jbusres.2014.11.027

[B11] Chen J. V. Su B. C. Widjaja A. E. (2016). Facebook C2C social commerce: a study of online impulse buying. *Decis. Support Syst.* 83 57–69. 10.1016/j.dss.2015.12.008

[B12] Chen Y. Lu Y. Wang B. Pan Z. (2019). How do product recommendations affect impulse buying? An empirical study on WeChat social commerce. *Inf. Manage.* 56 236–248.

[B13] Chin W. W. Marcolin B. L. Newsted P. R. (2003). A partial least squares latent variable modeling approach for measuring interaction effects: results from a Monte Carlo simulation study and an electronic-mail emotion/adoption study. *Inf. Syst. Res.* 14 189–217. 10.1287/isre.14.2.189.16018 19642375

[B14] Dean D. H. (1999). Brand endorsement, popularity, and event sponsorship as advertising cues affecting consumer pre-purchase attitudes. *J. Advert.* 28 1–12. 10.1080/00913367.1999.10673585

[B15] Deephouse D. Carter S. (2005). An examination of differences between organizational legitimacy and organizational reputation. *J. Manage. Stud.* 6 3–23.

[B16] Djafarova E. Bowes T. (2021). ‘Instagram made me buy it’: generation Z impulse purchases in fashion industry. *J. Retailing Consum. Serv.* 59:102345. 10.1016/j.jretconser.2020.102345

[B17] Doney P. M. Cannon J. P. (1997). An examination of the nature of trust in buyer-seller relationships. *J. Mark.* 61 35–51. 10.1177/002224299706100203

[B18] eMarketer (2021). *Over 45% of China’s Digital Shoppers will buy via Livestream in 2023.* Available onine at: http://www.emarketer.com/content/over-45-of-china-s-digital-shoppers-will-buy-via-livestream- 2023 (accessed October 30, 2021).

[B19] Fei M. Tan H. Peng X. Wang Q. Wang L. (2021). Promoting or attenuating? An eye-tracking study on the role of social cues in e-commerce livestreaming. *Decis. Support Syst.* 142:113466.

[B20] Fornell C. Larcker D. F. (1981). Structural equation models with unobservable variables and measurement error: algebra and statistics. *J. Mark. Res.* 18 382–388. 10.1177/002224378101800313

[B21] Galinsky A. D. Gruenfeld D. H. Magee J. C. (2003). From power to action. *J. Pers. Soc. Psychol.* 85 453–466. 14498782 10.1037/0022-3514.85.3.453

[B22] Gao X. Xu X.-Y. Tayyab S. M. U. Li Q. (2021). How the live streaming commerce viewers process the persuasive message: an ELM perspective and the moderating effect of mindfulness. *Electron. Commer. Res. Appl.* 49:101087. 10.1016/j.elerap.2021.101087

[B23] Gulfraz M. B. Sufyan M. Mustak M. Salminen J. Srivastava D. K. (2022). Understanding the impact of online customers’ shopping experience on online impulsive buying: a study on two leading E-commerce platforms. *J. Retailing Consum. Serv.* 68:103000.

[B24] Guo Y. Zhang K. Wang C. (2022). Way to success: understanding top streamer’s popularity and influence from the perspective of source characteristics. *J. Retailing Consum. Serv.* 64:102786. 10.1016/j.jretconser.2021.102786

[B25] Hair J. F. Hult G. T. M. Ringle C. M. Sarstedt M. (2017). *A Primer on Partial Least Squares Structural Equation Modeling (PLS-SEM)*, 2nd Edn. Thousand Oaks, CA: Sage Publishing.

[B26] Hair J. F. Sarstedt M. Hopkins L. Kuppelwieser V. G. (2014). Partial least squares structural equation modeling (PLS-SEM) an emerging tool in business research. *Eur. Bus. Rev.* 26 106–121. 10.1108/EBR-10-2013-0128

[B27] He Y. Oppewal H. (2018). See how much we’ve sold already! Effects of displaying sales and stock level information on consumers’ online product choices. *J. Retailing* 94 45–57. 10.1016/j.jretai.2017.10.002

[B28] Hopkins E. (2022). Machine learning tools, algorithms, and techniques in retail business operations: consumer perceptions, expectations, and habits. *J. Self Gov. Manage. Econ.* 10 43–55. 10.22381/jsme1012023

[B29] Huang L.-T. (2016). Flow and social capital theory in online impulse buying. *J. Bus. Res.* 69 2277–2283. 10.1016/j.jbusres.2015.12.042

[B30] Jacoby J. (2002). Stimulus-organism-response reconsidered: an evolutionary step in modeling (consumer) behavior. *J. Consum. Psychol.* 12 51–57. 10.1207/153276602753338081

[B31] Jang S. Chung J. (2021). What drives add-on sales in mobile games? The role of inter-price relationship and product popularity. *J. Bus. Res.* 124 59–68. 10.1016/j.jbusres.2020.11.025

[B32] Jeffrey S. A. Hodge R. (2007). Factors influencing impulse buying during an online purchase. *Electron. Commer. Res.* 7 367–379. 10.1007/s10660-007-9011-8

[B33] Jiang H. Liu B. Sun P. (2018). The influence of power on consumer behavior and its theoretical explanation. *Adv. Psychol. Sci.* 26 156–168. 10.3724/SP.J.1042.2018.00156

[B34] Jin S.-A. A. Phua J. (2014). Following celebrities’ tweets about brands: the impact of twitter-based electronic word-of-mouth on consumers’ source credibility perception, buying intention, and social identification with celebrities. *J. Advert.* 43 181–195. 10.1080/00913367.2013.827606

[B35] Kang K. Lu J. Guo L. Li W. (2021). The dynamic effect of interactivity on customer engagement behavior through tie strength: evidence from live streaming commerce platforms. *Int. J. Inf. Manage.* 56:102251.

[B36] Kao K. C. Hill S. R. Troshani I. (2021). A cross-country comparison of online deal popularity effect. *J. Retailing Consum. Serv.* 60:102402. 10.1016/j.jretconser.2020.102402

[B37] Karimov F. P. Brengman M. (2014). An examination of trust assurances adopted by top Internet retailers: unveiling some critical determinants. *Electron. Commer. Res.* 14 459–496. 10.1007/s10660-014-9148-1

[B38] Kim J. H. (2018). Effect of brand popularity as an advertising cue on tourists’ shopping behavior. *J. Destination Mark. Manag.* 10 78–86. 10.1016/j.jdmm.2018.07.001

[B39] Klieštik T. Kovalova E. Lãzãroiu G. (2022). Cognitive decision-making algorithms in data-driven retail intelligence: consumer sentiments, choices, and shopping behaviors. *J. Self-Gov. Manage. Econ.* 10 30–42. 10.22381/jsme1012022

[B40] Kotler P. (1973). Atmospherics as a marketing tool. *J. Retailing* 49 48–64.

[B41] Koufaris M. (2002). Applying the technology acceptance model and flow theory to online consumer behavior. *Inf. Syst. Res.* 13 205–223. 10.1287/isre.13.2.205.83 19642375

[B42] Ladhari R. Massa E. Skandrani H. (2020). YouTube vloggers’ popularity and influence: the roles of homophily, emotional attachment, and expertise. *J. Retailing Consum. Serv.* 54:102027. 10.1016/j.jretconser.2019.102027

[B43] Lee J. Aggarwal A. Rafieian H. Korschun D. (2020). Do consumers use tipping to monitor service? Role of power and embarrassment. *J. Retailing Consum. Serv.* 56:102159. 10.1016/j.jretconser.2020.102159

[B44] Li R. Lu Y. Ma J. Wang W. (2021). Examining gifting behavior on live streaming platforms: an identity-based motivation model. *Inf. Manage.* 58:103406.

[B45] Li Y. Li X. Cai J. (2021). How attachment affects user stickiness on live streaming platforms: a socio-technical approach perspective. *J. Retailing Consum. Serv.* 60:102478. 10.1016/j.jretconser.2021.102478

[B46] Liu Y. Li H. Hu F. (2013). Website attributes in urging online impulse purchase: an empirical investigation on consumer perceptions. *Decision Support Syst.* 55 829–837. 10.1016/j.dss.2013.04.001

[B47] Lo P.-S. Dwivedi Y. K. Tan G. W.-H. Ooi K.-B. Aw E. C.-X. Metri B. (2022). Why do consumers buy impulsively during live streaming? A deep learning-based dual-stage SEM-ANN analysis. *J. Bus. Res.* 147 325–337.

[B48] Lu B. Chen Z. (2021). Live streaming commerce and consumers’ purchase intention: an uncertainty reduction perspective. *Inf. Manage.* 58:103509. 10.1016/j.im.2021.103509

[B49] Luo X. Andrews M. Song Y. Aspara J. (2014). Group-buying deal popularity. *J. Mark.* 78 20–33.

[B50] MacKenzie S. B. Podsakoff P. M. (2012). Common method bias in marketing: causes, mechanisms, and procedural remedies. *J. Retailing* 88 542–555. 10.1016/j.jretai.2012.08.001

[B51] Magnini V. P. Karande K. Singal M. Kim D. (2013). The effect of brand popularity statements on consumers’ purchase intentions: the role of instrumental attitudes toward the act. *Int. J. Hospitality Manage.* 34 160–168. 10.1016/j.ijhm.2013.02.010

[B52] Mehrabian A. Russell J. A. (1974). *An Approach to Environmental Psychology.* Cambridge (MA): MIT Press.

[B53] Meilatinova N. (2021). Social commerce: factors affecting customer repurchase and word-of-mouth intentions. *Int. J. Inf. Manage.* 57:102300. 10.1016/j.ijinfomgt.2020.102300 35245323

[B54] Milliman R. E. (1982). Using background music to affect the behavior of supermarket shoppers. *J. Mark.* 46 86–91. 10.1177/002224298204600313

[B55] Mou J. Shin D. (2018). Effects of social popularity and time scarcity on online consumer behaviour regarding smart healthcare products: an eye-tracking approach. *Comput. Hum. Behav.* 78 74–89. 10.1016/j.chb.2017.08.049

[B56] Nica E. Sabie O.-M. Mascu S. Luţan (Petre) A. G. (2022). Artificial intelligence decision-making in shopping patterns: consumer values, cognition, and attitudes. *Econ. Manage. Financl. Marke.* 17 31–43. 10.22381/emfm17120222

[B57] Nichols B. S. (2012). The development, validation, and implications of a measure of consumer competitive arousal (CCAr). *J. Econ. Psychol.* 33 192–205. 10.1016/j.joep.2011.10.002

[B58] Parboteeah D. V. Valacich J. S. Wells J. D. (2009). The influence of website characteristics on a consumer’s urge to buy impulsively. *Inf. Syst. Res.* 20 60–78. 10.1287/isre.1070.0157 19642375

[B59] Park H. J. Lin L. M. (2020). The effects of match-ups on the consumer attitudes toward internet celebrities and their live streaming contents in the context of product endorsement. *J. Retailing Consum. Serv.* 52:101934. 10.1016/j.jretconser.2019.101934

[B60] Pelau C. Dabija D.-C. Ene I. (2021). What makes an AI device human-like? The role of interaction quality, empathy and perceived psychological anthropomorphic characteristics in the acceptance of artificial intelligence in the service industry. *Comput. Hum. Behav.* 122:106855. 10.1016/j.chb.2021.106855

[B61] Pera R. Viglia G. Furlan R. (2016). Who am I? How compelling self-storytelling builds digital personal reputation. *J. Interact. Mark.* 35 44–55. 10.1016/j.intmar.2015.11.002

[B62] Rook D. W. (1987). The buying impulse. *J. Consum. Res.* 14 189–199. 10.1086/209105

[B63] Rucker D. D. Galinsky A. D. Dubois D. (2012). Power and consumer behavior: how power shapes who and what consumers value. *J. Consum. Psychol.* 22 352–368. 10.1016/j.jcps.2011.06.001

[B64] Sengupta A. S. Balaji M. S. Krishnan B. C. (2015). How customers cope with service failure? A study of brand reputation and customer satisfaction. *J. Bus. Res.* 68 665–674. 10.1016/j.jbusres.2014.08.005

[B65] Singh J. Crisafulli B. Quamina L. T. Xue M. T. (2020). ‘To trust or not to trust’: the impact of social media influencers on the reputation of corporate brands in crisis. *J. Bus. Res.* 119 464–480. 10.1016/j.jbusres.2020.03.039

[B66] Spangenberg E. R. Crowley A. E. Henderson P. W. (1996). Improving the store environment: do olfactory cues affect evaluations and behaviors? *J. Mark.* 60 67–80. 10.1177/002224299606000205

[B67] Spence M. (1973). Job market signaling. *Q. J. Econ.* 87 355–374. 10.2307/1882010 28848463

[B68] Statista. (2021). *Live Streaming E-Commerce in China.* Available online at: https://www.Statista.com/study/82597/live-commerce-in-china/ (accessed July 1, 2022)

[B69] Stern H. (1962). The significance of impulse buying today. *J. Mark.* 26 59–62. 10.1177/002224296202600212

[B70] Su L. Swanson S. R. Chinchanachokchai S. Hsu M. K. Chen X. (2016). Reputation and intentions: the role of satisfaction, identification, and commitment. *J. Bus. Res.* 69 3261–3269.

[B71] Sun Y. Shao X. Li X. Guo Y. Nie K. (2019). How live streaming influences purchase intentions in social commerce: an IT affordance perspective. *Electron. Commer. Res. Appl.* 37:100886.

[B72] Swain S. D. Hanna R. Abendroth L. J. (2006). How time restrictions work: the roles of urgency, anticipated regret, and deal evaluations. *Adv. Consum. Res.* 33 523–525.

[B73] Swani K. Milne G. R. (2017). Evaluating Facebook brand content popularity for service versus goods offerings. *J. Bus. Res.* 79 123–133. 10.1016/j.jbusres.2017.06.003

[B74] Teubner T. Graul A. (2020). Only one room left! How scarcity cues affect booking intentions on hospitality platforms. *Electron. Commer. Res. Appl.* 39:100910. 10.1016/j.elerap.2019.100910

[B75] van Herpen E. Pieters R. Zeelenberg M. (2009). When demand accelerates demand: trailing the bandwagon. *J. Consum. Psychol.* 19 302–312. 10.1016/j.jcps.2009.01.001

[B76] Wei C. Liu M. W. Keh H. T. (2020). The road to consumer forgiveness is paved with money or apology? The roles of empathy and power in service recovery. *J. Bus. Res.* 118 321–334. 10.1016/j.jbusres.2020.06.061

[B77] Wells J. D. Parboteeah V. Valacich J. S. (2011). Online impulse buying: understanding the interplay between consumer impulsiveness and website quality. *J. Assoc. Inf. Syst.* 12 32–56. 10.17705/1jais.00254

[B78] Whitson J. A. Liljenquist K. A. Galinsky A. D. Magee J. C. Gruenfeld D. H. Cadena B. (2013). The blind leading: power reduces awareness of constraints. *J. Exp. Soc. Psychol.* 49 579–582.

[B79] Wongkitrungrueng A. Assarut N. (2020). The role of live streaming in building consumer trust and engagement with social commerce sellers. *J. Bus. Res.* 117 543–556. 10.1016/j.jbusres.2018.08.032

[B80] Wongkitrungrueng A. Valenzuela A. Sen S. (2018). The cake looks yummy on the shelf up there: the interactive effect of retail shelf position and consumers’ personal sense of power on indulgent choice. *J. Retailing* 94 280– 295. 10.1016/j.jretai.2018.07.001

[B81] Wu I.-L. Chen K.-W. Chiu M.-L. (2016). Defining key drivers of online impulse purchasing: a perspective of both impulse shoppers and system users. *Int. J. Inf. Manage.* 36 284–296. 10.1016/j.ijinfomgt.2015.11.015

[B82] Wu Y. Xin L. Li D. Yu J. Guo J. (2021). How does scarcity promotion lead to impulse purchase in the online market? A field experiment. *Inf. Manage.* 58:103283.

[B83] Yang K. Kim H. M. Tanoff L. (2020). Signaling trust: cues from Instagram posts. *Electron. Commer. Res. Appl.* 43:100998. 10.1016/j.elerap.2020.100998

[B84] Yi Y. Gong T. Lee H. (2013). The impact of other customers on customer citizenship behavior. *Psychol. Mark.* 30 341–356. 10.1002/mar.20610

[B85] Zhang M. Liu Y. Wang Y. Zhao L. (2022). How to retain customers: understanding the role of trust in live streaming commerce with a socio-technical perspective. *Comput. Hum. Behav.* 127:107052.

